# Impact on children of a parent with ALS: a case-control study

**DOI:** 10.3389/fpsyg.2015.00288

**Published:** 2015-03-17

**Authors:** Vincenzo Calvo, Francesca Bianco, Enrico Benelli, Marco Sambin, Maria R. Monsurrò, Cinzia Femiano, Giorgia Querin, Gianni Sorarù, Arianna Palmieri

**Affiliations:** ^1^Department of Philosophy, Sociology, Pedagogy and Applied Psychology, University of PadovaPadova, Italy; ^2^Department of Medical, Surgical, Neurological, Metabolic and Aging Sciences, Second University of NaplesNaples, Italy; ^3^Department of Neurosciences, University of PadovaPadova, Italy

**Keywords:** ALS, chronic medical condition (CMC), parents-children, problem behavior, Rorschach

## Abstract

**Background:** Numerous studies have explored how patients and their caregivers cope with amyotrophic lateral sclerosis (ALS), but the literature completely lacks research on the psychological impact of the disease on patients’ children. The aim of our study was to investigate the emotional and psychological impact of a parent with ALS on school-age children and adolescents in terms of problem behavior, adjustment, and personality characteristics.

**Methods:** The study involved 23 children (mean age = 10.62 years, six females) with a parent suffering from ALS, and both their parents. Children were matched for age, gender, and birth-order with a control group of children with healthy parents. They were administered the Youth Self Report (YSR) questionnaire and the Rorschach Comprehensive System, and their healthy parent completed the Child Behavior Checklist (CBCL).

**Results**: Findings clearly showed that, compared with controls, children with a parent who had ALS had several clinically significant adverse emotional and behavioral consequences, with emotional and behavioral problems, internalizing problems, anxiety and depressive symptoms. Children of a parent with ALS scored higher than controls for the Total Problems, Internalizing Problems, Anxious/Depressed and Withdrawn/Depressed scales in the YSR. A relevant percentage of children fell within the clinical range (42.9%) and borderline range (28.6%) for Internalizing Problems. The Rorschach CS confirmed the substantial impact of ALS in a parent on their offspring in terms of internalizing behavior and depression, with adjustment difficulties, psychological pain, and thought problems.

**Conclusion**: Our findings indicate that school-aged children and adolescents with a parent who has ALS are vulnerable and carry a substantially higher risk of internalizing behavior, depressive symptoms, and reactive problems than children with healthy parents. Families affected may need support to cope with such an overwhelming disease.

## Introduction

Amyotrophic lateral sclerosis (ALS) is a degenerative disease involving both upper and lower motor neurons, and leading relentlessly to progressive muscle atrophy and weakness, dysphagia and dysarthria. In most cases, patients die within 3–5 years of the disease’s onset, usually due to respiratory failure (Rowland and Shneider, 2001). Secondary symptoms that often affect patients and, indirectly, their caregivers in everyday life are pain (Pagnini et al., 2011), emotional lability (Palmieri et al., 2009), and sleep disturbance (Blackhall, 2012).

There is no effective cure for ALS, so medical treatment is focused on physical symptom management (Aoun et al., 2012). The impact of the disease on patients can be huge, particularly on the early stage after diagnosis, often causing anxiety, depression, anguish, and suicidal ideation (Palmieri et al., 2010; Pagnini et al., 2012).

Amyotrophic lateral sclerosis is overwhelming and challenging for caregivers and families as well. When Cipolletta and Amicucci (2014) interviewed members of the families of ALS patients who had died, they found that the disease was perceived as a death sentence for the patient on the one hand, and as a family illness on the other. The authors concluded that ALS produces both centrifugal and centripetal adjustment processes (i.e., creating a greater distance between some family members, but bringing others closer together) and has several profound consequences on everyday family life.

Patients with ALS have to cope with serious restrictions and changes in their daily life. They need assistance with eating and dressing, nursing care, and mobility, that is often provided by a family member, and the patient’s partner in nearly 80% of cases (Miller et al., 2000). The progressive nature of the disease gradually increases the patient’s dependence on the primary caregiver, who may sometimes spend more than 11 h a day with the patient (Krivickas et al., 1997). Family caregivers of ALS patients are deeply affected by the illness and often experience burden due to physical strain, emotional tension, personal and social limitations (Rabkin et al., 2000), anxiety and depression (Chiò et al., 2005; Pagnini et al., 2012). Significantly, such symptomatic expressions in caregivers are reportedly connected with the patients’ gradual loss of physical function (Gauthier et al., 2007; Pagnini et al., 2010).

Although many studies have explored how patients and their caregivers cope with ALS, the literature completely lacks any research on the specific psychological impact of such a devastating disease on the patients’ children. It is important to investigate this issue in the case of ALS because the consequences of a parent’s severe illness on their offspring can sometimes be dramatic, as already reported for a variety of diseases. In particular, the impact on children of a parent suffering from a chronic medical condition (CMC), i.e., a disease lasting at least 3 months and involving one or more apparata (Brown, 2006), such as cancer, HIV/AIDS, multiple sclerosis (MS), rheumatoid arthritis, brain damage, as well as others, has been well investigated.

According to the extant literature, a CMC in a parent involves the whole family and has a great impact on the parent’s children (Visser-Meily et al., 2005). Long-term behavioral problems have been described in such children (van de Port et al., 2007), and their consequences have been seen to continue for several years after the parent’s death (Wong et al., 2009). To give an example, Wong et al. (2009) examined the long-term impact of the childhood experience of a parent suffering from cancer and found that 44% of the individuals involved reported posttraumatic growth experiences in adulthood, while 59% reported experiencing adverse consequences, such as feelings of loss and void, concern for own health, negative changes in outlook on life, and negative impact on personal relationships.

More in general, children of an ill parent were reported to experience high levels of stress and to be at higher risk of health-related and social-emotional problems, such as somatic complaints, social isolation, excessive concern about becoming ill themselves, anxiety, depressive affects, and low self-esteem (Compas et al., 1994; Earley and Cushway, 2002; Faulkner and Davey, 2002; Ivarsson et al., 2002; Pedersen and Revenson, 2005; Flahault and Sultan, 2010; Sieh et al., 2010). A meta-analysis conducted by Sieh et al. (2010) showed that children growing up with a chronically ill parent were significantly more likely to have problem behavior than children of healthy parents. They were particularly at risk of internalizing behavior (anxiety, depression and withdrawal, and somatic complaints), but there was also evidence of more externalizing problems (aggressive and rule-breaking behavior) in the children with a chronically ill parent.

Among the studies on children with parents suffering from neurological CMCs, the case of MS has been the most thoroughly investigated (Arnaud, 1959; Blackford, 1992; Cross and Rintell, 1999; Steck et al., 2005; Diareme et al., 2006; Pakenham and Bursnall, 2006; Coles et al., 2007; Kalb, 2007; Steck et al., 2007; Yahav et al., 2007; Ehrensperger et al., 2008; Pakenham and Cox, 2008; Bogosian et al., 2010; Morley et al., 2011; Razaz et al., 2014). These studies have highlighted the strong impact of a parent with neurological disease on a child’s psychological adaptation. Some recurrent developmental outcomes were identified, such as high levels of depression, anxiety, and low self-esteem, with changing roles, a heightened sense of responsibility and internalizing disorders.

Given these premises, the present study had three main objectives. First, we aimed to verify whether school-age children and adolescents who have a parent with ALS are more at risk of problem behavior, as measured by self-report questionnaires, than children of healthy parents. Consistently with the extant literature on CMCs in parents, we expected children with a parent who had ALS to display more internalizing and/or externalizing problems than controls. Second, we aimed to explore the psychological adjustment of children with a parent suffering from ALS by administering a performance-based task, the Rorschach test, scored using the Comprehensive System (CS; Exner, 2003; Exner and Edberg, 2005). Like Flahault and Sultan (2010), who used the Rorschach CS to examine how children adapted to having a parent with cancer as opposed to other illnesses, we aimed to integrate the information deriving from self-report questionnaires with a performance-based task. The Rorschach CS is a validated and reliable performance-based task that generates information of which the subject being tested may be unaware (Flahault and Sultan, 2010). The third aim of the study was to explore how parents perceived their children’s behavior and competences. In particular, we wanted to test the consistency and agreement between the parents’ and their children’s reported internalizing and externalizing problem behavior. The purpose to investigate the agreement among parents and children originates from the fact that previous research has shown that parents may underreport the problems of their children (Sourander et al., 1999). The demands and difficulties of the disease may make it arduous for a parent to recognize the needs of the children and to provide reliable information about their psychological functioning. The presence of discrepancies between parent and children reports may have some important implications for clinical practice. If children report more problems than parents do, it is likely that some of them do not receive appropriate psychological help because their difficulties remain unnoticed by adults (Sourander et al., 1999).

## Materials and Methods

### Participants

The study group consisted of 23 children with a parent suffering from ALS. The mean age of the children was 10.62 years (*SD* = 3.98 years, range: 5–17 years), six were females (26.1%), 17 were male (73.9%). The children belonged to 12 families in which one parent had ALS and the healthy spouse was the patient’s main caregiver. The ill parent was the mother in five families, the father in seven.

Twenty-three children matched with the study group for age, gender, and birth-order formed a control group. They were recruited from the general population of children attending primary or secondary schools in the same area as the study group.

All participants were Caucasian and none of their parents were single. The participants’ demographic details are shown in **Table 1**.

**Table 1:** Demographic characteristics of the study sample and control group.

Parents’ characteristics
	Ill parent (n = 12)	Spouse (n = 12)
Mothers (*n,* %)	5 (41.7%)	7 (58.3%)
Fathers (*n,* %)	7 (58.3%)	5 (41.7%)
Age (mean, SD)	45.12 (6.5)	42.75 (3.2)
Age range (years)	31.5–52.5	38.0–47.7
Duration of illness, years (mean, SD)	6.93 (4.2)	
ALSFRS (mean, SD)	26 (8.4)	
Limb onset (*n,* %)	11 (91.7%)	
Bulbar onset (*n,* %)	1 (8.3%)	
**Children’s characteristics**
	**Study group (n = 23)**	**Control group (n = 23)**

Gender (*n,* %)		
Female	6 (26.1%)	6 (26.1%)
Male	17 (73.9%)	17 (73.9%)
Age (mean, SD)	10.62 (4.0)	10.74 (3.9)
Age range (years)	5.0–17.6	5.0–17.5
Number of children participating in the study per family (*n,* percent)		
1	5 (41.7%)	18 (90%)
2	3 (25%)	1 (5%)
3	4 (33.3%)	1 (5%)

*ALSFRS, Amyotrophic lateral sclerosis functional rating scale.*

### Instruments and Measures

#### Children’s Emotional/Behavioral Problems and Competences

Children aged 11 years and over (*n* = 28) were administered the Youth Self Report (YSR; Achenbach and Rescorla, 2001) and, for all the children the healthy parent completed the Child Behavior Checklist (CBCL; Achenbach and Rescorla, 2001).

The YSR is a self-administered questionnaire, part of the Achenbach System of Empirically Based Assessments (ASEBAs; Achenbach and Rescorla, 2001), developed to assess emotional and behavioral problems in children and adolescents between the ages of 11 and 18 years. The YSR includes 118 items rated on a 3-point scale ranging from 0 (not true) to 2 (very true). The instrument provides measures for Internalizing, Externalizing, and Total Problems, and eight syndrome scales, designated as Anxious/Depressed, Withdrawn/Depressed, Somatic Complaints, Social Problems, Thought Problems, Attention Problems, Rule-Breaking Behavior, and Aggressive Behavior, with higher scores indicating more severe symptoms. The YSR also yields a measure of social competences, the Total Competence scale (comprising the Activities, Social, and School subscales), that assesses the amount and quality of the youth’s involvement in sports, organizations, jobs and chores, social relationships, and school performance.

The CBCL is a rating scale, also part of the ASEBA, and it is the parallel form of the YSR for parents. It provides parent-reported information on a broad range of emotional and behavioral difficulties in a child during the previous 6 months. Like the YSR, the CBCL includes 118 items for measuring the eight syndrome scales and the three general dimensions of Internalizing, Externalizing, and Total Problems, plus 20 competence items (referring to the child’s participation in hobbies, games, sports, jobs, chores, friendship, and activities).

As recommended by Achenbach and Rescorla (2001), we used raw scores for all comparisons involving the YSR and CBCL scores to take the full range of variation in these scales into account. The scores for Internalizing, Externalizing, and Total Problems were also converted into *T* scores to ascertain clinical, borderline, and normal ranges: *T* score below 60 = normal; between 60 and 63 = borderline; and 64 or above = clinical (Achenbach and Rescorla, 2001).

The CBCL and the Teacher’s Report Form in the ASEBA have shown good validity and reliability characteristics in the Italian population (Frigerio et al., 2004).

#### Personality Characteristics

The children’s personality characteristics were assessed using the Rorschach test, a performance-based task consisting of 10 official inkblots (five made with black ink on a white background, two with black and red ink on a white background, and three with multicolored ink on a white background). Forty-five Rorschach protocols were collected because one child in the study group refused to complete the Rorschach task. Exner’s CS was used to administer and interpret the Rorschach protocols (Exner, 2003; Exner and Edberg, 2005), and the ‘Structural Summary’ of the scores was produced using the ROR-SCAN software (Caracena, 2002). The Rorschach CS protocols were scored by two experienced clinical psychologists because it has been demonstrated that training and experience with administering and scoring this type of task can have a major effect on CS scores (Lis et al., 2007). The Rorschach CS has standardized procedures, a high inter-scorer reliability, adequate test–retest reliability and validity, and reference norms based on large samples of normative or pathological respondents (Weiner and Exner, 1991; Weiner, 2000).

To obtain inter-scorer reliability, 10 protocols set were selected at random and scored independently by the two judges. Inter-scorer reliability was calculated by percentage of agreement and iota for 10 response segments. Iota is chance-corrected reliability coefficient that is equivalent to Cohen’s kappa for a multivariable test scored by two or more raters (Janson, 2003, 2004). The percentages of agreement and iota values for response segments indicated satisfactory inter-scorer reliability (**Table 2**).

**Table 2:** Rorschach inter-scorer agreement on coding segments.

Variable	% Agreement	Iota (Kappa)
Whole responses	0.975	0.793
Location and space (two variables)	0.954	0.895
DQ (+, o, v/+, v)	0.894	0.732
Determinants (11 variables)	0.978	0.835
FQ (None, +, o, u, -)	0.673	0.512
Pairs	0.954	0.895
Contents (27 variables)	0.987	0.830
P	0.954	0.795
Z score	0.899	0.825
CS special scores (14 variables)	0.989	0.587

To prevent chance findings arising from multiple comparisons, we restricted the Rorschach variables analyzed in this study (Weiner, 1995), as in the method used by Flahault and Sultan (2010). Among the clusters belonging to the Structural Summary (Control and Management of Stress, Affects, Self-Perception, Interpersonal Relationships, Processing, Mediation, Ideation, and Psychopathological Constellations), we selected the Rorschach variables that had proved to be reliable markers of self-perception, affect modulation, individual adjustment, cognitive style, interpersonal functioning, emotional distress, anxiety, and depression (Weiner, 1998; Exner, 2003). As in Flahault and Sultan (2010), self-esteem was operationalized using the EGO index (reflection and pair responses); desirable self-image or pleasure in interaction with others using the GHR responses (Good Human Responses); physical preoccupations using the An + Xy contents (anatomy and radiography); emotional distress with the D score (tolerance of stress, control); anxiety and affects due to stress with the SumY (shading-diffusion) and m (object movement); and negative thoughts about the self and the world with the Vista (shading-vista), MOR (morbid content), SumC’ (achromatic color), and DEPI (depression index) variables.

Several indexes were used to assess respondents’ ideational processes and cognitive resources, namely variables regarding reality testing (X-%, distorted form; X+%, conventional form; XA%, very good form quality percent; Xu%, usual form), and variables regarding thought problems (i.e., Sum6, Number of Special Score; WSum6, Weighted Sum of Special Score; Level 2, Raw Number of Lv2 Special Score). We also used the intellectualizing index (2AB + [Art + Ay]) to examine variables that might reflect defensive mechanisms, such as the tendency to take refuge in fantasy (Ma/Mp), or to minimize emotional experiences. All these variables were analyzed as frequencies (considering the number of responses), and/or as categories when the frequency exceeded a cutoff validated in the CS.

Finally, we analyzed the Psychopathological Constellation indexes. When positive, these indexes point to the presence of depressive symptoms (DEPI), the risk of suicidal behavior (S-CON, Suicide Constellation), inadequate coping abilities (CDI, Coping Deficit Index), obsessive cognitive processing (OBS, Obsessive Style Index), paranoid thoughts (HVI, Hypervigilance Index), and thought disturbance (PTI, Perceptual Thinking Index).

### Procedure

Patients with possible, probable, or definite ALS – according to the El Escorial criteria for its clinical diagnosis (Ross et al., 1998; Brooks et al., 2000) – and their families were recruited through the Neurosciences Department of the University of Padova and the Department of Medical, Surgical, Neurological, Metabolic and Aging Sciences of the Second University of Naples (SUN). All ALS patients recruited in our study were living at home with their families. A psychotherapist, experienced with ALS, informed both patients and their caregivers about the purposes of the study by telephone. Patients who agreed to participate were included in an experimental protocol that involved a home visit, during which a clinical psychologist trained in the use of the Rorschach CS administered the various tools to the patient’s children and healthy spouse. Each home visit took ∼90 min. The study was approved by the Ethics Committee of Padova University. Written informed consent was obtained from all parents participating in the study.

### Data Analysis

We compared the study and control groups for the measures derived from the YSR, CBCL, and Rorschach CS, using the non-parametric statistic of the Mann–Whitney *U* test for continuous variables and Pearson’s chi square for categorical variables. The Mann–Whitney *U* test was preferred to other parametric statistics because non-parametric tests require few if any assumptions about the shapes of the underlying population distributions and are more robust with small sample size (Siegel and Castellan, 1988). The criterion adopted for statistical significance was α = 0.05 (two-tailed exact significance) and the effect sizes of differences were calculated as Cohen’s (1988) *d* and classified, according to Cohen’s guidelines, as small (*d* = 0.20), medium (*d* = 0.50), or large (*d* = 0.80). Cohen’s *d* values of 1.30 and above were classed as very large (Sullivan and Feinn, 2012).

To check the consistency between the YSR completed by the children in the study group and the CBCL completed by their parents, we used Pearson’s product–moment correlations to assess relative agreement (*T*-scores), and intraclass correlation coefficients (ICCs) to assess pairwise agreement between informants (absolute agreement). Pearson’s correlation coefficient indicates a poor agreement when lower than 0.30, a moderate agreement between 0.30 and 0.50, and a good agreement when higher than 0.50 (Cohen, 1988). An ICC suggests poor agreement below 0.40, moderate to good agreement between 0.40 and 0.75, and excellent agreement above 0.75 (Novella et al., 2001). Wilcoxon Signed Ranks Tests were conducted to assess differences in the mean *T* scores obtained by the children in the study group and their healthy parents.

## Results

### Children’s Emotional/Behavioral Problems and Competences

First we examined whether the children with a parent who had ALS differed from the controls in terms of emotional and behavioral problems and competences, as measured by the YSR (*n* = 28). The study group scored significantly higher than the control group for: Anxious/Depressed (*U* = 35.0, *z* = -2.92, *p* = 0.003), Withdrawn/Depressed (*U* = 41.0, *z* = -2.65, *p* = 0.007), Internalizing (*U* = 31.0, *z* = -3.08, *p* = 0.001), and Total Problems (*U* = 41.0, *z* = -2.62, *p* = 0.008). The differences for Aggressive Behavior (*U* = 58.5, *z* = -1.84, *p* = 0.066) neared statistical significance, while no differences emerged for Externalizing Problems or any of the other syndrome and social competence scales (**Table 3**). The effect sizes relating to the scales with significant differences were large (*d* > 0.8), and for Internalizing Problems Cohen’s *d* was very large (> 1.30; Sullivan and Feinn, 2012).

**Table 3:** Comparison of emotional and behavioral problems and competences (YSR) between children with and without a parent suffering from ALS.

	Study group (*N* = 14)		Control group (*N* = 14)					Cohen’s *d*
Scale	*M*	SD	*M*	SD	*U*	*z*	*P*	
Anxious/Depressed	8.86	3.30	5.00	2.88	35.0	-2.92	0.003**	1.25
Withdrawn/Depressed	4.43	2.34	2.14	1.70	41.0	-2.65	0.007**	1.12
Somatic Complaints	4.50	4.16	2.79	1.67	81.0	-0.79	0.442	0.54
Social Problems	4.50	2.95	2.86	2.14	64.0	-1.58	0.119	0.64
Thought Problems	3.57	3.32	1.86	1.41	67.5	-1.43	0.158	0.67
Attention Problems	6.36	3.52	4.64	1.98	78.0	-0.93	0.364	0.60
Rule-Breaking Behavior	2.00	0.88	2.36	2.90	80.5	-0.822	0.423	-0.17
Aggressive Behavior	8.07	3.73	5.64	3.10	58.5	-1.84	0.066	0.70
Internalizing	17.79	7.03	9.93	4.71	31.0	-3.08	0.001**	1.31
Externalizing	10.07	3.91	8.00	5.55	61.5	-1.68	0.095	0.43
Total problems	47.93	17.94	30.57	13.25	41.0	-2.62	0.008**	1.10

Activities	6.74	2.98	8.03	2.50	77.0	-0.97	0.345	-0.47
Social	6.86	1.57	7.56	1.92	72.5	-1.18	0.248	-0.40
School	2.94	2.36	2.32	0.45	94.0	-0.19	0.863	0.36
Total Competence	15.93	3.37	17.91	3.94	71.0	-1.24	0.227	-0.54

** *p* < 0.01.

Analyzing the scores for Internalizing, Externalizing, and Total Problems in terms of their clinical relevance, we found 6 (42.9%) of 14 children with a parent who had ALS were in the clinical range on the Internalizing scale, 4 (28.6%) were borderline, and only 4 (28.6%) were in the normal range. Conversely, none of the 14 children in the control group was in the clinical range, 2 were borderline, and 12 were in the normal range. The difference between the two groups was statistically significant (χ^2^ [2, *N* = 28] = 10.67, *p* = 0.004).

No such differences emerged between the groups in terms of their distribution in the Externalizing (χ^2^ [2, *N* = 28] = 3.04, *ns*), and Total problem (χ^2^ [2, *N* = 28] = 4.17, *ns*) scales.

### Children’s Personality Characteristics

The personality characteristics (derived from the Rorschach CS) of the children in the two groups were compared using the Mann–Whitney *U* test and chi-square statistics: the statistically significant results are presented in **Tables 4** and **5**, respectively.

**Table 4:** Mann–Whitney *U* test comparing continuous variables in the Rorschach Comprehensive System (CS) between children with and without a parent suffering from ALS.

		Study group (*N* = 22)		Control group (*N* = 23)					
CS indexes	Interpretation (after Exner, 2003)	*M*	SD	*M*	SD	*U*	*z*	*p*	Cohen’s *d*
SumV	Self-disgust, shame, emotional distress, guilt	0.73	0.70	0.04	0.21	113.0	-3.92	<0.001***	1.34
FC	Controlled emotional experiences	0.64	0.85	1.96	1.67	126.0	-3.01	0.002**	-0.99
An	Preoccupations concerning body, health or illness	1.16	0.25	0.66	0.14	183.5	-1.79	0.078	2.47
MOR	Negative or blemished features about the self and the world	1.73	1.39	0.65	1.56	104.5	-3.57	<0.001***	0.73
GHR	Positive self-image	1.64	1.62	3.04	1.99	146.5	-2.45	0.013*	-0.77

XA%		64.23	16.52	91.39	9.01	25.0	-5.19	<0.001***	-2.04
*X*--*%*	Distorted perceptions	34.00	15.34	7.52	7.43	18.0	-5.35	<0.001***	2.20
Xu%		36.41	14.11	59.00	12.32	57.5	-4.44	<0.001***	-1.71

Sum6		4.64	5.20	1.00	1.31	103.0	-3.49	<0.001***	-0.48
Level 2		0.82	1.30	0.04	0.21	158.5	-2.96	0.003**	0.84
Alog	Thought disturbance	1.32	2.15	0.17	0.65	127.0	-3.42	<0.001***	0.72
CTM		0.86	1.21	0.00	0.00	138.0	-3.59	<0.001***	1.01
WSum6		19.95	23.88	2.74	4.47	88.0	-3.82	<0.001***	1.00
M-		1.27	1.72	0.26	0.45	157.0	-2.48	0.013*	0.80

2AB + (Art + Ay)	Minimizing emotional experiences by intellectualizing	1.59	1.47	0.83	1.07	169.5	-1.99	0.049*	1.39

PTI	Perceptual-thinking index	1.95	1.50	0.09	0.29	57.0	-4.92	<0.001***	1.72
DEPI	Depression index	4.23	1.19	3.57	0.95	169.0	-1.97	0.050	0.61

*p < 0.05, **p < 0.01, ***p < 0.001.

**Table 5:** Chi-square comparison of categorical variables in the Rorschach Comprehensive System (CS) between children with and without a parent suffering from ALS.

		Study group (*N* = 22)		Control group (*N* = 23)			
CS indexes	Interpretation (according to Exner, 2003)	Freq.	*%*	Freq.	*%*	Chi-square	p
SumV > 0	Self-disgust, shame, emotional distress, and guilt	13	59.1	1	4.3	15.72	<0.001***
GHR < PHR	Healthy and adaptive understanding of others	17	77.3	8	34.8	8.22	0.004**
*MOR > 1*	Negative or blemished features about the self and the world	10	45.5	4	17.4	4.13	0.042*

XA% > 0.90		0	100	16	69.5	23.75	<0.001***
X-% > 0.14	Distorted perceptions	21	95.5	5	21.7	25.05	<0.001***
X-% > 0.25		16	72.7	2	8.7	19.21	<0.001***

M > 0		12	54.5	6	26.1	3.79	0.051
Level 2 > 0	Thought disturbance	9	40.9	1	4.3	8.70	0.003**
Ma < Mp		0	0	4	17.4	4.20	0.040*

PTI > 2	Index of disturbed thinking and distorted perceptions	9	40.9	0	0	11.76	0.001**
DEPI > 4	Higher probability of subclinical or clinical depression	10	45.5	4	17.4	4.13	0.042*
CDI > 3, DEPI > 4	Tendency toward affective problems	3	13.6	0	0	3.76	0.053

*p < 0.05, **p < 0.01, ***p < 0.001.

The children with a parent suffering from ALS had several variables that, interpreted in accordance with the CS, were indicative of a less adequate psychological functioning than the children in the control group. They showed signs of more self-disgust, shame, emotional distress, and guilt (SumV, SumV > 0), more negative or blemished features about the self and the world (MOR, MOR > 1), more preoccupation with the body, health or illness (An nearing statistical significance, *p* = 0.078), less well controlled or modulated emotional experiences (FC), undesirable self-image (a lower GHR than controls), and less healthy and adaptive understanding of others (GHR < PHR). They had less adequate reality testing results (XA%, XA% > 0.90), more distorted perceptions (X-%) and signs of serious mediational impairments (X-% > 0.14, X-% > 0.25). These children with a parent suffering from ALS also showed more signs of thought disturbance (Sum6, Level 2, Level 2 > 0, Alog, CTM, WSum6, M-).

The children in the study group revealed other differences vis-à-vis the control children too. They had less unconventional, uncommon, or creative views (Xu%), they more frequently exhibited human movements indicative of general mental abilities, including planning, imagination, and empathy (M > 0). They also tended to minimize emotional experiences by intellectualizing [2AB + (Art + Ay)]. None of the children with a parent suffering from ALS used fantasies as an integral part of their defense system (Ma < Mp).

Among the pathological constellations of the Rorschach CS, the DEPI (i.e., the intensity of depressive affects) was significantly higher in the study group, and significantly more children in the study group (10 of 22, 45.5%) than in the control group (4 of 23, 17.4%) came above the clinical threshold (DEPI > 4) indicating a higher probability of subclinical or clinical depression. Three children in the study group, but none in the control group, had a DEPI > 4 associated with a CDI ≥ 4 (i.e., a positive CDI): this association – which neared statistical significance (*p* = 0.053) – is interpreted as a tendency toward affective problems. Similarly, the index of disturbed thinking and distorted perceptions, Perceptual-Thinking Index (PTI), was significantly higher in the study group than in the control group, and 9 of the 22 children in the study group who completed the Rorschach Test (40.9%) had a PTI ≥ 3, a categorical cutoff that has been found correlated with high thought disorder scores in other tests (Hilsenroth et al., 2007).

The children in the study group did not differ significantly from the controls in the other psychopathological constellations, i.e., the S-CON, the HVI, and the OBS.

### Children’s Emotional/Behavioral Problems and Competences as Perceived by their Parents

We compared the two groups of children in terms of their emotional and behavioral problems and competences as reported by their healthy parents using the CBCL (*n* = 46 for global scores [Internalizing, Externalizing, and Total Problems]; *n* = 40 for the subscales). The statistical comparisons yielded no significant differences between the two groups for the syndrome and general scales of the CBCL: Anxious/Depressed (*U* = 185.0, *z* = -0.41, *ns*), Withdrawn/Depressed (*U* = 155.0, *z* = -1.24, *ns*), Somatic Complaints (*U* = 141.0, *z* = -1.67, *ns*), Social Problems (*U* = 181.5, *z* = -0.51, *ns*), Thought Problems (*U* = 197.5, *z* = -0.07, *ns*), Attention Problems (*U* = 199.5, *z* = -0.01, *ns*), Rule-Breaking Behavior (*U* = 138.0, *z* = -1.75, *ns*), Aggressive Behavior (*U* = 144.0, *z* = -1.54, *ns*), Internalizing (*U* = 196.0, *z* = -1.51, *ns*), Externalizing (*U* = 189.5, *z* = -1.66, *ns*), and Total Problems (*U* = 211.0, *z* = -1.18, *ns*).

On the other hand, parents of the children in the study group returned significantly lower scores than parents of children in the control group for all competence scales of the CBCL except School (*U* = 190.5, *z* = -0.26, *ns*). More in detail, children with an ALS parent were described as being significantly less competent in: activities (*U* = 81.0, *z* = -3.22, *p* =0.001), Social (*U* = 109.5, *z* = -2.45, *p* = 0.013), and Total Competence (*U* = 88.5, *z* = -3.02, *p* = 0.002).

Then we applied Pearson’s correlation and ICCs to examine the agreement between the scores obtained by the children in the study group and their healthy parents. Moderate to high Pearson’s correlations were found for Internalizing, Externalizing, and Total Problems (*r* = 0.358, *r* =0.491, *r* = 0.597, respectively). The ICCs generated similar results (ρ = 0.345, ρ = 0.462, ρ = 0.595, respectively). Wilcoxon Signed Ranks Tests identified no significant differences between the healthy parents’ and their children’s assessments of the latter’s Internalizing (*z* = -0.72, *ns*), Externalizing (*z* = -1.19, *ns*), and Total problem T scores (*z* = -1.64, *ns*).

## Discussion

Our study investigated the emotional and psychological impact of having a parent with ALS on school-age children and adolescents, in terms of adjustment, problem behavior, and personality characteristics, because many studies have explored how patients and caregivers cope with ALS, but none have investigated the psychological impact of the disease on patients’ children.

Our findings clearly showed that, compared with a control group of offspring of healthy parents, children who have a parent with ALS experience several emotional and behavioral adverse consequences in terms of global emotional and behavioral problems, internalizing problems, anxiety, and depression, and clinically significant indexes relating to their personality characteristics.

The first aim of our study was to see whether children of a parent with ALS are more at risk of problem behavior than a control group of children with healthy parents. Specifically, children of a parent with ALS reported more emotional and behavioral problems in the YSR, as measured by the Total Problems scale, and more Internalizing problems than the control group. They also had significantly higher scores on two syndrome scales in the internalizing domain of the YSR (Anxious/Depressed and Withdrawn/Depressed), and scores that neared a statistically significant difference on the Aggressive behavior scale of the YSR. Notably, all the corresponding effect sizes were either large (for Anxious/Depressed, Withdrawn/Depressed, and Total Problems) or very large (for Internalizing Problems), indicating that the differences between the scores obtained by the two groups were very clear. A substantial percentage of children with an ALS parent fell within the clinical (42.9%) and borderline (28.6%) ranges for Internalizing problems according to the YSR cutoffs; the difference vis-à-vis the control group was statistically significant.

On the whole, our results are in line with the extant literature suggesting that children can be strongly affected by having a chronically ill parent, especially in terms of internalizing problems (Visser et al., 2005; Sieh et al., 2010) and related symptoms, such as depression, anxiety, and withdrawal (Sieh et al., 2010). ALS in a parent appears to have the same effects on children as any other chronic and/or terminal disease, but the large effect sizes identified in our sample seem to indicate that children with a parent who has ALS are at substantially greater risk of internalizing behavior than it would seem from the literature on the children of parents with other CMCs. For instance, the meta-analysis conducted by Sieh et al. (2010) yielded a significant overall effect size for self-reported internalizing behavior, indicating that children with a chronically ill parent had more internalizing problems than other children, but the mean effect size was small (*d* = 0.25; 95% CI [0.18, 0.31]). The very large effect size (*d* = 1.31) for self-reported internalizing problem in our sample led us to hypothesize that ALS in parents may have a significantly worse fallout on their children’s adaptation than other chronic illnesses. While this issue requires further investigation, two preliminary one-sided outlier tests, carried out to detect whether the effect size for self-reported internalizing problem in our sample is an outlier with respect to the distribution of effect scores reported by Sieh et al. (2010), seemed to confirm this hypothesis (Dixon’s *Q*-test:* p* = 0.048; Grubb’s test: *p* = 0.017).

Having a parent with ALS did not increase the scores for somatic complaints in our study group, as reported in other studies concerning CMCs, and MS in particular. Arnaud (1959) found higher levels of body concern in the children of parents with MS, and Pakenham and Bursnall (2006) likewise reported a greater somatization. The latter authors suggested that, in accordance with the social learning model, the children of parents with MS may use somatization to model their parent’s illness behavior (Pakenham and Bursnall, 2006). In the light of this interpretation, we speculate that our study children did not express body concern because they were unable to model their parent’s illness behavior, possibly because ALS symptoms are too frightening.

Contrary to our expectations, we found no significant differences between our study and control groups in terms of externalizing problems (as reported in many other studies on children with parents suffering from CMCs). To clarify this result, we should consider that the Externalizing score in the YSR consists of two clinical subscales for Rule-Breaking Behavior and Aggressive Behavior. While there were clearly no differences between our two groups of children for the first subscale, on the second the study group revealed higher levels of aggressive behavior than the controls, and the difference neared statistical significance with a medium effect size (*p* = 0.066, *d* = 0.70); increasing the size of the sample would probably make this difference statistically significant. As a possible explanation for this result, there is the interesting suggestion from Sieh et al. (2012) that internalizing problems may buffer against the development of externalizing problems. Children and adolescents with high levels of anxiety, withdrawal, and fearfulness are probably less likely to engage in risk-taking behavior (Sieh et al., 2012).

To sum up, we found that children and adolescents with a parent suffering from ALS had high levels of internalizing problems but few or no externalizing problems. Looking at the broader picture, having a parent with ALS globally affected the children’s adjustment – given their significantly higher YSR scores for Total Problems. But analyzing the YSR subscales comprising the Total Problems score showed that the study group’s specific reaction pattern was due essentially to internalizing problems and, to a lesser extent, to aggressive behavior. Visser et al. (2005) had likewise reported a prevalence of internalizing problems (withdrawal, somatic complaints, and anxiety/depression) over externalizing problems (rule-breaking or aggressive behavior) in children with parents suffering from cancer.

More in general, our findings are in line with other reports on the profound effect of having a parent with a CMC on a child’s functioning, though the combinations of internalizing and externalizing problems may vary. Diareme et al. (2006), for instance, described both internalizing problems (withdrawal, somatic complaints, anxiety/depression) and externalizing problems (rule-breaking and aggressive behavior) in children and adolescents who had a parent with MS. Similarly, Rodrigue and Houck (2001) found an amalgam of internalizing, externalizing, social, identity, and thought problems in a group of children who had parents with various health conditions. In contrast, as Visser et al. (2005) noted, the children of divorced parents reportedly experience more externalizing than internalizing problems (Hetherington and Stanley-Hagan, 1999). We agree that different stressors seem to trigger problems in different areas, and a parent with ALS seems more likely to induce children to turn inward emotionally rather than to exhibit outward-directed behavioral problems (Visser et al., 2005).

The second aim of our study was to test the impact of ALS in parents on their offspring’s personality characteristics using the Rorschach CS, in much the same way as Flahault and Sultan (2010) studied adaptation in children with a parent suffering from cancer. Our results reinforce and extend the general picture emerging from the YSR.

First, the Rorschach CS confirmed the substantial impact of ALS in a parent in terms of internalizing behavior and depression in their offspring. The DEPI of the Rorschach CS, which indicates the intensity of depressive affects, was significantly higher in our study group than in the control group, and a significant proportion of the former children (45.5%) scored above the clinical threshold pointing to a higher likelihood of subclinical or clinical depression. It is worth emphasizing that the percentage of children above the clinical threshold for depression in the Rorschach CS (45.5%) was very similar to the percentage of children identified by the YSR as having internalizing problems above the clinical cut-off (42.9%). In other words, the self-report and projective measures converged in indicating that more than 40% of children with a parent suffering from ALS have internalizing-depressive problems.

Second, the structural summary of the children in our study group (obtained with the Rorschach CS) revealed several indicators of difficult adjustment and psychological pain. In their projective responses, these children showed more self-disgust, shame, emotional distress, and guilt. They also experienced more negative or blemished features about the self and the world, an undesirable self-image, less well controlled or modulated emotional experiences, and a less healthy and adaptive understanding of others. The Rorschach CS also generated projective indexes of preoccupations with the body, health and illness that did not emerge from the YSR. A psychodynamic interpretation of this inconsistency might be that these children were worried and scared about their parent’s body, health or illness (as indicated by the Rorschach projections), and they coped with their concern by unconsciously adopting defense mechanisms such as repression, denial, or dismissal. In fact, previous research has shown that physical evidence of a parent’s illness and treatment (e.g., a mother’s hair loss due to treatment for breast cancer; Forrest, 2006) may have a great impact on children in terms of the distress they experience, and that the impact of a parent’s disease is higher, the more their visible symptoms are severe (Kennedy and Lloyd-Williams, 2009).

Overall, the results obtained with the Rorschach CS are particularly congruent with the extant literature on children whose parents have advanced or terminal disease. Kennedy and Lloyd-Williams (2009) investigated how children cope with the distress they experience when a parent is diagnosed with advanced cancer. Children were described as being very distressed by their parent’s diagnosis and having concerns relating to their parent’s and their own health. Maintaining the appearance of normality and distraction were the prevailing coping strategies used by such children, while having a more limited social activity and greater responsibilities were the most manifest life changes (Kennedy and Lloyd-Williams, 2009).

Another finding emerging from the Rorschach CS in the present study is that our study group had higher scores in several indexes relating to thought problems. The children in the study group proved less adequate in reality testing, they had more distorted perceptions and signs of serious mediational impairment, along with direct evidence of thought disturbance emerging from several scores included in the PTI (Exner, 2000). It is important to bear in mind that the PTI (the result of a thorough revision of the SCZI index) is not meant to be a specific diagnostic indicator of schizophrenia, thought disorder, and psychotic processes (Smith et al., 2001). It should be considered as a tool that can alert clinical psychologists to the possibility of disturbed thinking or cognitive slippage (Smith et al., 2001), conditions that may be interpreted in several ways when found in children with a parent who has ALS.

In our opinion, these indexes should be seen as depending on traumatic events in the children’s history, possibly due to the stress associated with a parent’s illness. Consistently with this interpretation, Pynoos et al. (1996) reported that Rorschach CS protocols of traumatized adolescents revealed complex psychological profiles, partially similar to those found in our study group. In particular, they highlighted the presence of cognitive problems (revealed through non-normative responses on EA, X+%, Lambda, and F+%), intrusive fantasies and memory disruptions [Schizophrenic index (SCZI) and M-], and loss of self-esteem (Ego Index, MOR, DEPI, and V). In the same vein, Holaday (2000) collected and interpreted Rorschach protocols from children and adolescents diagnosed with posttraumatic stress disorder, finding high scores in the SCZI, DEPI, and CDI indexes, as well as in other variables. The author suggested that, when young victims of traumatic experiences cannot “make sense of what has happened to them, life becomes irrational, illogical, and confusing. Reality is no longer understood in the same way as it was before the trauma […] and the unexplainable, disorganized, and hurtful thoughts and feelings experienced by traumatized children and adolescents are revealed through their Rorschach protocols” (Holaday, 2000, p. 155).

Other studies have identified thought problems connected with post-traumatic experiences in the children of ill parents. Huizinga et al. (2005), for instance, studied the adolescent and young adult offspring of parents diagnosed with cancer, and found that the experience of cancer in a parent may lead to stress response symptoms (i.e., post-traumatic symptoms): in their sample, 21% of the sons and 35% of the daughters of parents diagnosed with cancer had clinically significant stress response symptoms in the form of intrusive thoughts and avoidance behavior, and their stress response symptoms were associated in turn with thought problems and attention problems. Sons and daughters with these clinical stress response symptoms both had significantly higher scores on thought problems and attention problems than adolescents in a norm group (boys also differed from the norm group for anxiety/depression; and girls for withdrawal, somatic complaints, and anxiety/depression). Notably, as the authors highlighted, the incidence of stress response symptoms among children with a parent who had cancer was higher than the incidence seen in other studies among children who experienced cancer themselves. This evidence “suggests that witnessing cancer in a family member may have a more profound impact on a child than being a cancer victim oneself” (Huizinga et al., 2005, p. 293). We hypothesize that witnessing such a devastating terminal illness as ALS in a parent may well produce similar, if not worse effects in terms of the child’s stress response and post-traumatic symptoms, with consequent thought problems. However, further research into this aspect is needed.

The third aim of our study was to explore how parents see their children in terms of problem behavior and competences. According to previous literature, it is common for children and adolescents to report experiencing more problems than their parents perceive in them (Stanger and Lewis, 1993; Sourander et al., 1999; Žukauskienė et al., 2004; Watson et al., 2006). Contrary to expectations, the parents and children in our study group showed a moderate to good agreement in reporting problem behavior, and they did not differ in terms of mean internalizing, externalizing, and total problem scores. It was only when compared with the control group that parental perceptions revealed some differences. Parents of the children in the study group awarded significantly lower scores in all competence scales of the CBCL except for School (i.e., Activities, Social, and Total Competence). Specifically, children in the study group were described by their healthy parents as being less capable in a variety of activities (e.g., sports) and social interactions (e.g., friendships) than other children and adolescents of similar age, whereas their school adjustment appeared to be unaffected. In our opinion, these findings indicate that the healthy parent in families with a parent suffering from ALS were aware of their children’s difficult life experience and tended not to underestimate their problems. This awareness of the healthy parents and, more in general, the quality of their emotional availability, is a key issue that warrants further investigation in ALS families. Numerous studies concerning other diseases have indicated that the quality of the emotional availability of the healthy parent may compensate for the inattentiveness of the other parent who is ill. For the children to have a significant relationship with the healthy parent may be fundamental to their psychological adjustment (Davies and Windle, 1997; Leinonen et al., 2003).

In short, our study shows that school-aged children and adolescents who have a parent with ALS are vulnerable and liable to suffer mainly in terms of internalizing behavior and depressive symptoms, and thought problems as a reaction to their stressful condition. Strikingly, about 40% of our sample had scores in the clinical range for internalizing/depressive problems. The healthy parents seemed to confirm this picture and are probably aware of the impact of their spouse’s ALS on their children.

Several limitations apply to this study. First, the sample size was not large enough to allow for any reliable generalization of our results. Second, the age range of the children considered was rather wide. The small number of children considered also prevented us from assessing any influence of moderators relating to the children (e.g., age and gender) or the situation (e.g., features of the parent’s illness, the patient’s neuropsychological and psychopathological profile, parental functioning, and socioeconomic status) that could help to explain the differences in problem behavior between the study group and the controls. A last, potential limitation lies in that we considered more than one child from the same family, thus violating the assumption of statistical independence of our observations. Having said that, we must remember that ALS is a relatively rare disease and it is consequently very difficult to obtain a large number of participants.

Despite its limitations, to the best of our knowledge this exploratory study is the first to address the impact of ALS in a parent on a child’s adjustment. We believe that the severe psychological consequences of having a parent with ALS emerging from the present study mean that the families affected may need support to cope with such an overwhelming disease.

## Author Contributions

Design and conceptualization of the study: AP, GS, VC. Coding of data: AP, FB. Statistical analysis and interpretation of the data: VC. Drafting the manuscript: VC, FB. Revising the manuscript for intellectual content: VC, FB, EB, MS, MM, CF, GQ, GS, AP. Final approval of the version to be published: VC, FB, EB, MS, MM, CF, GQ, GS, AP. Agreement to be accountable for all aspects of the work in ensuring that questions related to the accuracy or integrity of any part of the work are appropriately investigated and resolved: VC, FB, EB, MS, MM, CF, GQ, GS, and AP.

## Conflict of Interest Statement

The authors declare that the research was conducted in the absence of any commercial or financial relationships that could be construed as a potential conflict of interest.
